# Synthesis, Thermal Stability, and Emission Properties of Eu_2_O_3_ and Tm_2_O_3_ Doped Halide Phosphate Glasses Based on the P_2_O_5_–ZnO–BaF_2_–LiCl–CdO System

**DOI:** 10.3390/ma19132706

**Published:** 2026-06-23

**Authors:** Reem D. Alshehri, Ali M. Alshehri, Badriah Sultan, Zahrah S. A. Almutawah, Khalid I. Hussein, Mohammed S. Alqahtani, Bozena Burtan-Gwizdala, Manuela Reben, El Sayed Yousef

**Affiliations:** 1 Physics Department, Faculty of Science, King Khalid University, AlQura’a, Abha P.O. Box 906, Saudi Arabia; 444817190@kku.edu.sa (R.D.A.); amshehri@kku.edu.sa (A.M.A.); bsoltan@kku.edu.sa (B.S.); zalmutawah@kku.edu.sa (Z.S.A.A.); 2 Department of Radiological Sciences, College of Applied Medical Sciences, King Khalid University, AlQura’a, Abha P.O. Box 906, Saudi Arabia; mosalqhtani@kku.edu.sa (M.S.A.); ayousf@kku.edu.sa (E.S.Y.); 3 Central Labs, King Khalid University, AlQura’a, Abha P.O. Box 906, Saudi Arabia; 4 Department of Physics, Cracow University of Technology, ul. Podchorazych 1, 30-084 Cracow, Poland; burtan_bozena@wp.pl; 5 Faculty of Materials Science and Ceramics, AGH University of Krakow, al. Mickiewicza 30, 30-059 Kraków, Poland; manuelar@agh.edu.pl

**Keywords:** phosphate glass, thermal stability, energy gap, linear refractive index, emission cross section, physical parameters, visible color

## Abstract

**Highlights:**

- The PZBLC–Tm^3+^ glass demonstrates enhanced optical performance, with a gain bandwidth (GBW = σ_em_ × Δλ_eff_) calculated as 16.7 × 10^−26^ cm^3^.
- The PZBLC–Eu^3+^ glasses are promising candidates for red visible-light emission under 395 nm excitation.
- The PZBLC–Tm^3+^ glasses, excited at 357 nm, exhibit broadband near-infrared (NIR) emission suitable for optical amplification.
- These glasses emit blue light, making them useful as standard light sources.

**Abstract:**

The PZBLC glass system, with the molar composition 40P_2_O_5_–30ZnO–10BaF_2_–18LiCl–2.0CdO (mol%), was fabricated and subsequently doped with Eu_2_O_3_ and Tm_2_O_3_ using a melt-quenching technique. The thermal stability (ΔT), glass transition temperature (T_g_), and linear refractive indices of the fabricated glass were evaluated. The spectroscopic parameters Ω_2_, Ω_4_, and Ω_6_, and the measured visible and near-infrared photoluminescence at the excitation wavelength depend on the type of rare-earth ions in the doped glasses and were estimated. The lifetimes of the relevant transition levels and the gain bandwidths (σ_em_ × Δλ_eff_) of the fabricated glasses were evaluated. The PZBLC–Eu^3+^ glass, excited at 395 nm, exhibits an intense, high-purity red emission, whereas the PZBLC–Tm^3+^ glass, excited at 357 nm, shows a strong blue emission. The fabricated glasses are promising candidates as a solid source for visible-light emission with a high emission cross-section prepared by a low-cost technique.

## 1. Introduction

Rare-earth (RE) ion–doped glass is a highly promising host material due to its characteristic visible emissions, cost-effectiveness, straightforward fabrication, and improved thermal stability [1]. It has been widely applied across a range of optical technologies, including lasers, white-light-emitting diodes (W-LEDs), and optical amplifiers [2]. Over the past few decades, considerable research has been devoted to understanding the luminescence behavior and underlying mechanisms of various rare-earth (RE) ion-doped glass systems, including $Eu^{3+}$, $Er^{3+}$, $Tm^{3+}$, $Sm^{3+}$, $Tb^{3+}$, $Dy^{3+}$, $Nd^{3+}$, and $Yb^{3+}$ doped glasses [3,4,5,6,7,8,9,10]. Among the various RE ions, Eu^3+^ is regarded as one of the most significant activators in luminescent materials due to its relatively simple energy-level configuration, sharp and well-defined emission bands in the UV–Vis region, and intense red emission [11]. The luminescence of Eu^3+^ ions is largely independent of the crystal-field environment and is primarily governed by intra-configurational f–f transitions [12]. The electric-dipole transition near 612 nm (^5^D_0_ → ^7^F_2_) is sensitive to the host matrix ionicity and local symmetry, whereas the magnetic-dipole transition around 592 nm (^5^D_0_ → ^7^F_1_) remains essentially unaffected by changes in the surrounding environment. Consequently, the intensity ratio between magnetic-dipole and electric-dipole transitions is commonly used as a sensitive indicator of structural variations [13]. In this regard, Eu^3+^ ions play an essential role in luminescent materials, and their characteristic pure red emission has been extensively employed in spectral probes, white-light-emitting diodes (W-LEDs), and display technologies [14]. Meanwhile, trivalent thulium (Tm^3+^) ions exhibit emission over a broad spectral range, extending from the near-infrared (NIR) to the ultraviolet (UV) region. Tm^3+^—doped glasses have therefore gained considerable interest for several important reasons. Notably, the emission band centered at approximately 1460 nm, corresponding to the ^3^H_4_ → ^3^F_4_ transition, shows strong potential for use in fiber-optic amplifiers designed for telecommunication applications. The low-loss transmission window of optical fibers (1400–1600 nm) enables spectral extension into the S-band amplifier region, which lies on the shorter-wavelength side of the conventional erbium-doped fiber amplifier C-band (1530–1570 nm). Another emission band, centered near 1800 nm and associated with the ^3^F_4_ → ^3^H_6_ transition, is well suited for the development of lasers for medical applications as well as for atmospheric and chemical sensing [15,16,17]. Furthermore, the significant ^1^D_2_ → ^3^F_4_ transition of the Tm^3+^ ion produces blue emission around 450 nm through both down-conversion and up-conversion processes [18,19], making it valuable for high-capacity optical data storage technologies. It is well established that the luminescent behavior of glasses depends strongly on the chemical composition, structure, and overall characteristics of the host matrix. Among various glass systems, phosphate glass is regarded as an excellent host for RE-ion doping because of its favorable optical properties, moderate phonon energy, low melting temperature, low refractive index and dispersion, and its ability to accommodate high dopant concentrations [20]. However, the relatively poor thermal stability of pure phosphate glass restricts its practical use. To overcome this limitation, additional oxides are introduced to enhance structural stability.

ZnO functions as a network modifier within the glass system, decreasing hygroscopicity while improving mechanical strength. Incorporating zinc oxide into phosphate glasses lowers phonon energy, enhances transparency in the UV–visible range, and increases the solubility of rare-earth ions in the host matrix [2,8,21,22]. Studies by Zheng et al. [23] and Oueslati-Omrani et al. [24] demonstrated that ZnO can significantly improve the chemical durability of phosphate glass. It may also behave as a network former by generating [ZnO_4_] tetrahedral units or forming P–O–Zn linkages through ionic cross-linking, thereby strengthening and stabilizing the glass network. The CdO was intentionally introduced to modify the local phosphate network and improve the optical performance of the glass matrix. In phosphate glasses, CdO can act as a network modifier/intermediate, promoting the formation of non-bridging oxygens and altering the glass connectivity through Cd–O interactions. These structural changes may influence the local symmetry, phonon energy, and polarizability of the glass network, which are key factors governing luminescence efficiency and non-radiative relaxation processes [1,2].

Compared with the previously investigated CdO-free matrix, the incorporation of Cd^2+^ ions provides a more polarizable environment and may improve thermal stability and emission characteristics without significantly disturbing glass formation. Therefore, the introduction of CdO represents a targeted compositional modification aimed at tuning the structural and spectroscopic properties of the glass system, which strengthens the novelty of the present work [25,26].

For fluorescence and laser applications, dehydration of phosphate glasses—i.e., reducing the OH content—is essential. This is typically achieved through several methods, including the incorporation of chlorides or fluorides [27,28]. The addition of halides such as F^−^ and Cl^−^ enhances the phosphate glass matrix by increasing rare-earth ion solubility and creating multiple local environments for electric-dipole transitions [29].

As a result, hybrid glass structures can exhibit modified fluorescence line shapes along with altered radiative and nonradiative transition rates. Moreover, these halide ions help suppress OH^−^ incorporation into lattice sites. The presence of F^−^ and Cl^−^ also increases the ionicity and reduces the covalency of the glass network, leading to a wider optical band gap compared with purely oxide-based phosphate glasses [30].

The novelty of our work lies in the development of a phosphate-based PZBLC singly doped with rare-earth ions, namely Eu^3+^ or Tm^3+^, in separate glass compositions. This system exhibits tunable visible emission (red from Eu^3+^ and blue from Tm^3+^) alongside broadband near-infrared (NIR) luminescence, enhanced thermal stability, and promising characteristics for wavelength-division-multiplexed (WDM) optical amplification. Importantly, this study establishes a comprehensive correlation between the glass structure, spectroscopic parameters, and luminescence behavior, providing new insights into the design of multi-functional rare-earth-doped glasses for advanced photonic applications. F^−^, Cl^−^, Eu^3+^, and Tm^3+^ were incorporated into phosphate-zinc glasses to enhance both their luminescence and thermal properties. The optical energy gap and linear refractive index of the fabricated glasses were calculated using multiple methods. Additionally, visible and near-infrared (NIR) emission spectra and the decay lifetimes of the relevant transition levels were measured under specific excitation wavelengths. The results demonstrate that these fabricated glasses are promising candidates for advanced photonic applications.

## 2. Materials and Methods

### 2.1. Materials and Glass Preparation

**Figure 1:**
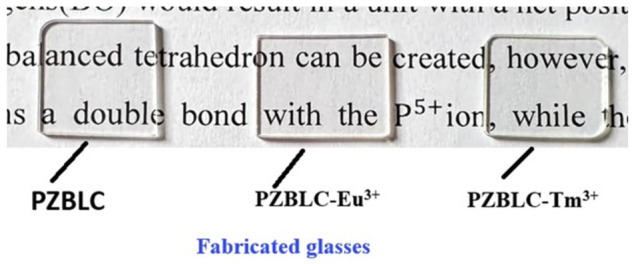
Fabricated glasses PZBLC, PZBLC-Eu^3+^ and PZBLC-Tm^3+^.

The studied glasses, with compositions 40P_2_O_5_–30ZnO–10BaF_2_–18LiCl–2.0Cd (PZBLC), 40P_2_O_5_–30ZnO–10BaF_2_–18LiCl–2.0Cd–35,000 ppm Eu_2_O_3_ (PZBLC–Eu), and 40P_2_O_5_–30ZnO–10BaF_2_–18LiCl–2.0Cd–35,000 ppm Tm_2_O_3_ (PZBLC–Tm), exhibited high homogeneity and transparency and were found to be non-hygroscopic (see Figure 1). Using the melt-quenching technique, the batches were melted in a muffle furnace at 1050 °C for 60 min, with continuous stirring in a platinum crucible to ensure homogeneity. Then the molten cast was poured into a copper mold and subsequently put in an annealing furnace, where the samples were kept at 430 °C. Annealing begins with a 2 h heat treatment to relieve strain.

The density of the glass samples was determined using a gas pycnometer (UltraPyc 1200e, Anton Paar QuantaTec Inc., Boynton Beach, FL, USA). The thermal properties of the obtained glasses, the optical absorption spectra of the prepared glasses, the luminescence decay curves, and the photoluminescence spectra were determined using the same equipment described in Article [31].

### 2.2. X-Ray Diffraction Analysis

**Figure 2:**
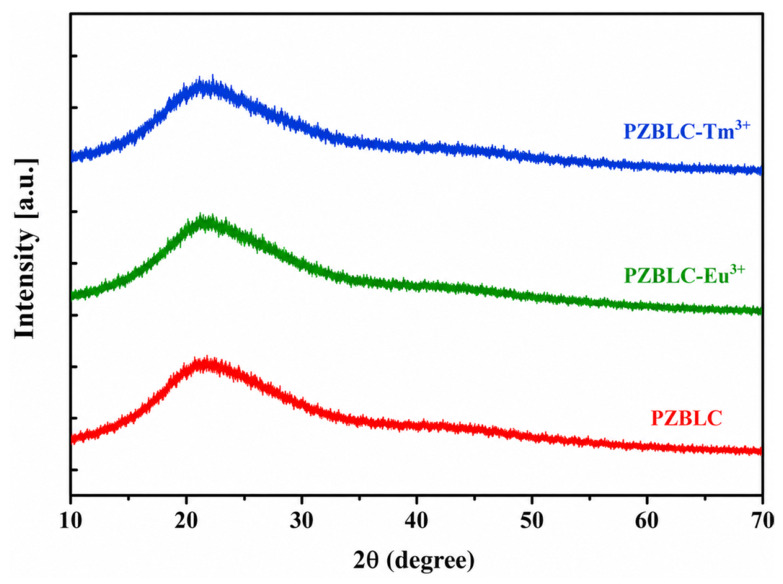
An X-ray diffraction (XRD) of the investigated glasses.

XRD analysis (Figure 2) was performed to confirm the amorphous nature of the studied glasses. The X-ray diffraction patterns exhibited broad diffuse halos without any sharp crystalline peaks, indicating the absence of long-range structural order and confirming the glassy nature of the samples. The XRD measurements were carried out using a X-ray diffraction patterns were recorded using a PANalytical X-ray diffractometer (PANalytical B.V., Almelo, The Netherlands) equipped with Cu Kα radiation.

### 2.3. Refractive Index

The refractive index dispersion of the prepared bulk glasses was investigated by means of spectroscopic ellipsometry (SE). Measurements were carried out using a Woollam M-2000 ellipsometer (Woollam Co. Inc., Lincoln, NE, USA), operating within the wavelength range of 200–1700 nm. Spectroscopic ellipsometry is a non-destructive optical technique that determines changes in the polarization state of light upon reflection from a sample surface. During the measurements, the ellipsometric parameters Ψ and Δ were recorded. These parameters provide a complete description of the polarization state of the reflected light and define the polarization ellipse in the plane perpendicular to the propagation direction.

Previous investigations have demonstrated that the refractive index dispersion of tellurite-based glasses can be accurately modeled using the Sellmeier equation (see Ref. [29] and references therein). Accordingly, the experimental data were fitted using the Sellmeier dispersion model, expressed as follows [1]:

(1) $$n=(A+\frac{B\lambda^{2}}{\lambda^{2}-C^{2}}-D\lambda^{2}{)}^{1/2}$$

where A represents the refractive index offset, B corresponds to the oscillator strength (amplitude), C denotes the resonance position, and D is associated with the infrared pole contribution.

## 3. Results and Discussion

### 3.1. Physical and Structural Properties

The molar volume, $V_{m\;}$; $\left[=(\sum X_{i}M_{i})\cdot \rho^{-1}\right]$, oxygen mole refraction, $V_{ο}$; $\left[=\;\left(\frac{\sum X_{i}M_{i}}{\rho }\;\right)\left(\frac{1}{\sum x_{i}n_{i}}\right)\;\right]$; $OPD=1000\;\left(\frac{\rho .\sum x_{i}n_{i}\;}{\sum X_{i}M_{i}}\right)$; were evaluated, where M is the molecular weight of the glass composition, ρ is the density, $x_{i}$ is the molar fraction, and $n_{i}$ is the number of oxygen atoms in each oxide. Table 1 lists the values of (ρ), ($V_{m\;}$), ($V_{ο\;}$), (OPD). The highest density was observed for the undoped PZBLC glass (ρ = 3.2573 g·cm^−3^), whereas the lowest density was recorded for the PZBLC–Eu^3+^ glass (ρ = 3.1046 g·cm^−3^), indicating a decrease in density upon incorporation of Eu_2_O_3_. The lowest values of molar volume (Vₘ) and oxygen packing density (Vₒ) were obtained for the PZBLC glass, while the highest values were estimated for the PZBLC–Tm^3+^ glass. The variations in density (ρ), molar volume (Vₘ), and oxygen packing density (Vₒ) do not exhibit linear behavior upon incorporation of the rare-earth ions Eu^3+^ and Tm^3+^. This nonlinearity may be associated with structural rearrangements within the glass network and possible changes in the local environment of the rare-earth ions. Furthermore, the oxygen packing density (OPD) decreases with increasing Tm_2_O_3_ content, indicating progressive structural rearrangement within the glass network.

This variation may suggest structural modifications that could involve changes in network connectivity and the distribution of oxygen species.

Furthermore, the increase in density may be consistent with stronger rare-earth–oxygen interactions and possible changes in network connectivity. This variation indicates that the structure becomes increasingly distorted due to the rise in the number of non-bridging oxygens (NBOs) generated by the cleavage of P–O–P bridges as Eu^3+^ and Tm^3+^ ions are progressively incorporated, leading to a decrease in the OPD values. However, the molar volume (Vₘ) is observed to increase with the addition of rare-earth oxides (Eu_2_O_3_ and Tm_2_O_3_) in the polyphosphate series, suggesting an increase in the average chain length of the metaphosphate glass network. Nevertheless, the magnitude of the variation in Vₘ is less significant than that observed for OPD. Lorentz-Lorenz the fallowing pattern was used to determine the molar refractivity, $R_{m}$, $=\left(\frac{n^{2}-\;1}{\;n^{2}+2}\;\right)V_{m\;}$, and molar polarizability$,\;\alpha_{m}\;=\frac{R_{m}}{2.52}$. The molar refractivity (Rₘ) and molar polarizability (αₘ), which are characteristic parameters of the investigated phosphate glasses, range from 17.15 to 21.08 m^3^/mol and from 6.8 to 8.04 Å^3^, respectively. Both parameters exhibit an inverse correlation with glass density, further supporting the occurrence of structural rearrangement within the phosphate network. The optical properties and thermal stability of these glasses are governed by several interrelated factors, including density, the polarizability of the first-neighbor coordinated anions, the coordination environment of the modifier cations, along with the inherent electronic polarizability of the oxide ions [24,32,33].

### 3.2. Thermal Properties

**Figure 3:**
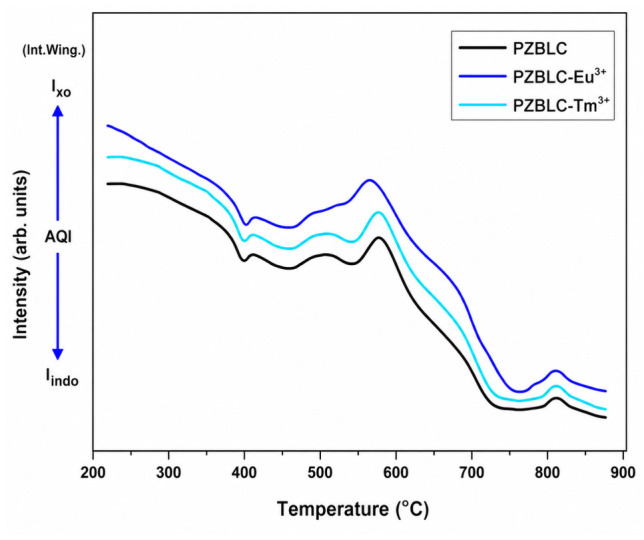
DTA curve of PZBLC, PZBLC-Eu^3+^, PZBLC-Tm^3+^.

As shown in Figure 3, the DTA curves were employed to evaluate the glass transition temperature (T_g_), the onset of crystallization temperature (T_x_), and the peak crystallization temperature (T_p_) of the synthesized glasses. The thermal stability of the glasses is often measured using the criteria ΔT= T_x_ − T_g_. As ΔT increases, anti-crystallization performance improves. The results are summarized in Table 2. The glass transition temperature (T_g_) ranges from 360 to 376 °C, while the crystallization temperature (T_x_) lies between 434 and 455 °C. The thermal stability parameter (ΔT = T_x_ − T_g_) varies from 74 to 79 °C. Finally, the specific heat capacity (C_p_) ranges from 0.237 to 0.361 J·mol^−1^·K^−1^. The high thermal stability indicates that the fabricated glasses exhibit lower crystal nucleation and a lower growth rate during fiber-drawing reheating. Furthermore, glasses studied have a suitable T_g_, indicating a high resistance to thermal damage. The increase in ΔT indicates that the glasses incorporating Eu^3+^/Tm^3+^ ions exhibit enhanced thermal stability, thereby providing stronger resistance to nucleation and crystallization compared to the undoped glass. In contrast, the rare-earth-free glass, which exhibits the lowest ΔT value, demonstrates reduced thermal stability and a greater tendency toward crystallization. Notably, the incorporation of Eu^3+^ and Tm^3+^ ions significantly affects the thermal properties of the 40P_2_O_5_–30ZnO–10BaF_2_–18LiCl–2.0Cd (PZBLC) glass system, leading to an increase in glass transition temperature (T_g_) and enhanced thermal stability.

This enhancement may be related to stronger rare-earth–oxygen interactions and structural rearrangements within the phosphate network. The higher field strength of the rare-earth ions may contribute to increased network rigidity, thereby strengthening the glass structure and inhibiting crystallization processes. This behavior can also be explained by factors related to the strength and nature of the chemical bonds formed with oxygen atoms. The incorporation of rare-earth ions leads to the formation of relatively strong and predominantly ionic Eu–O and Tm–O bonds in the PZBLC–Eu and PZBLC–Tm glasses, respectively. These stronger ionic interactions increase network rigidity and reduce structural mobility. As the rigidity of the glass network increases, a higher energy input is required to convert the rigid structure into a viscoelastic state, resulting in a glass transition at a raised temperature. Depending on the rare-earth oxide concentration and the composition of the glass matrix. RE^3+^ ions may enter the phosphate network and participate in the formation of P–O–RE–O–P linkages, although direct structural evidence is not available in the present study. These bonds, which may exhibit varying degrees of ionic or covalent character, act as ionic cross-links (P–O–RE), thereby reinforcing the glass structure and enhancing its thermal stability.

### 3.3. Optical and Spectroscopic Properties

**Figure 4:**
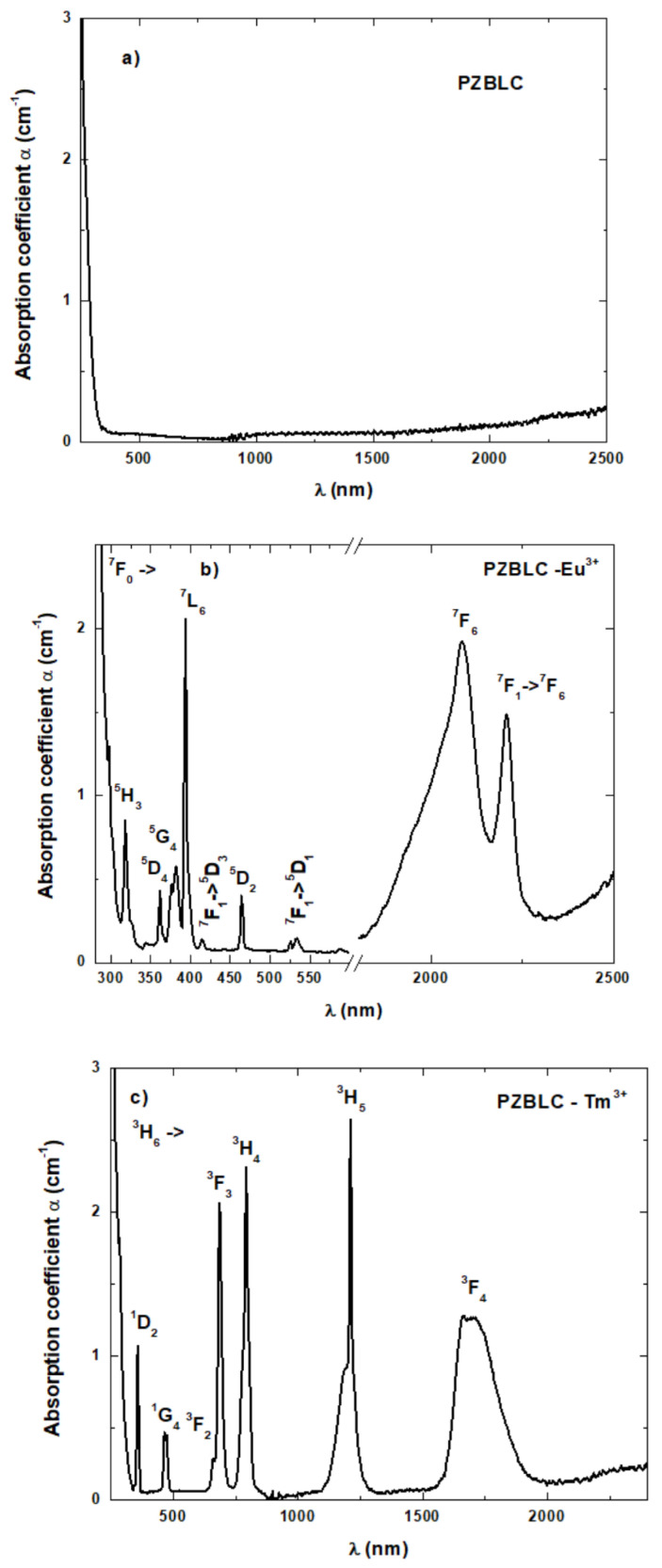
UV-Vis-NIR absorption spectra of fabricated glasses: (**a**) PZBLC, (**b**) PZBLC-Eu^3+^, (**c**) PZBLC-Tm^3+^.

Optical absorption measurements provide insight into the electronic transitions of glass materials. As shown in Figure 4a, the undoped PZBLC glass exhibits no significant absorption bands. In contrast (Figure 4b), the Eu_2_O_3_-doped glass (PZBLC–Eu^3+^) displays well-defined peaks corresponding to Eu^3+^ electronic transitions: ^7^F_0_ → ^5^D_4_ (362 nm), ^7^F_0_ → ^5^G_4_ (381nm), ^7^F_0_ → ^6^L_5_ (397 nm), ^7^F_1_ → ^5^L_6_ (414 nm), ^7^F_0_ → ^5^D_2_ (465 nm), ^7^F_1_ → ^5^D_1_ (533 nm), and ^7^F_1_ → ^7^F_6_ (2080 and 2206 nm). These observations are consistent with previous studies [7,12,16,21], confirming the introduction of Eu^3+^ ions into the phosphate glass network. The UV-Vis-NIR absorption spectra of fabricated glasses PZBLC-$Tm^{3+}$ (see Figure 4c) exhibits peaks attributed to excited transition levels from ^3^H_6_ → ^1^D_2_ (356 nm), ^3^H_6_ → ^1^G_4_ (470 nm), ^3^H_6_ → ^3^F_3_ (688 nm), ^3^H_6_ → ^3^H_4_ (787 nm), ^3^H_6_ → ^3^H_5_ (1210 nm) and ^3^H_6_ → ^3^F_4_ (1726 nm), which is similar to many other phosphate and fluorophosphate glasses doped with Tm^3+^ [34]. The incorporation of halide-containing oxides such as LiCl and BaF_2_, along with CdO, serves to enhance the ionic character of the host–ligand environment. This increases the crystal field strength of the Tm^3+ 3^H_4_ → ^3^F_4_ transition, thereby promoting near-infrared (NIR) emissions, as will be demonstrated later for the fabricated Tm^3+^-doped glass [23,35,36]. 

It is well established that, in insulators and semiconductors, the band gap represents the energy difference between the conduction band minimum and the valence band maximum. Based on the UV–Vis absorption spectra of all three samples, the optical band gap (E_opt_) was evaluated using the model proposed by Tauc [37], according to the relation:

(2) $${\left(\alpha h\nu \right)}^{r}=B(h\nu -E_{g})$$

**Figure 5:**
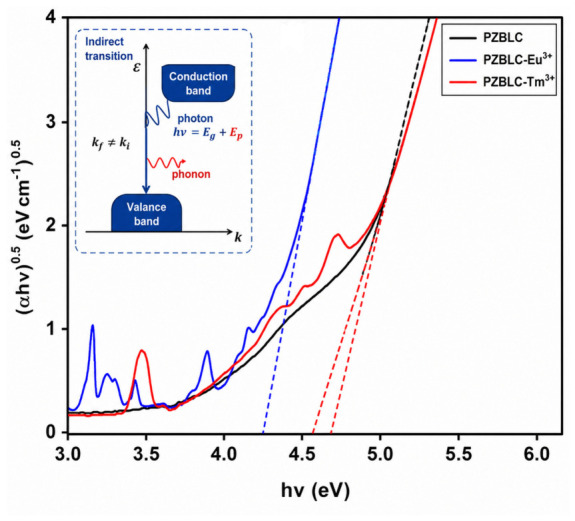
Variation of (αhυ)^0.5^ vs. hυ (eV) of fabricated glasses.

**Figure 6:**
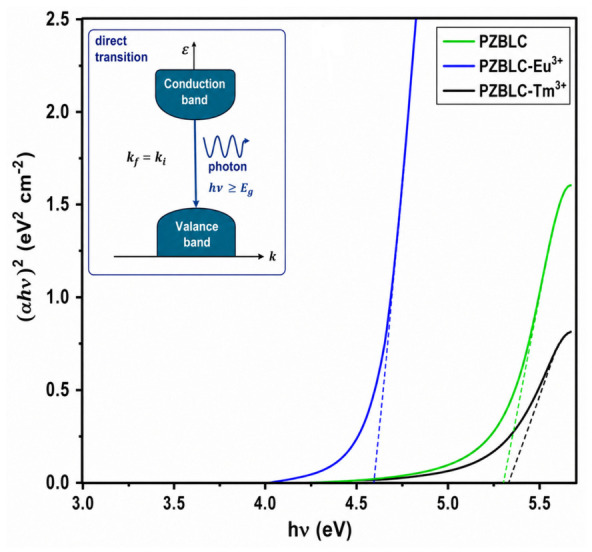
Variation of (αhυ)^2^ vs. (hυ) of fabricated glasses.

In this equation, B represents a constant often referred to as the band tailing parameter, r denotes the exponent related to the type of electronic transition, and E_g_ corresponds to the energy gap. The value of r indicates the nature of the electronic transition: for direct allowed transitions, r=2: for direct forbidden transitions: r=2/3: for indirect allowed transitions: r=1/2 and for indirect forbidden transitions: r=1/3. The value of the indirect optical energy gap,$\;E_{opt}^{In}$, and direct optical gap, $E_{opt}^{Di}$, were determined by using the absorption coefficient (*α*) and the photon energy (hν), which are illustrated in Figure 5 and Figure 6. The results obtained are listed in Table 3 and show that the value of $E_{opt}^{In}$ is in the range (4.236 to 4.749 eV) and $E_{opt}^{Di}$ within (4.639–5.320 eV). The glass (PZBLC) doped with $Eu^{3+}$ has the lowest value of both $E_{opt}^{In}$ and $E_{opt}^{Di}$ other than the (PZBLC) glass, which has the highest value. The undoped 40P_2_O_5_–30ZnO–10BaF_2_–18LiCl–2.0Cd glass exhibits the highest optical energy gap, whereas the glass doped with 35,000 ppm Eu_2_O_3_ (PZBLC–Eu^3+^) shows the lowest optical energy gap. Moreover, when the replacement of Eu^3+^ ions by Tm^3+^ led to an increase in the value of $E_{opt}^{In}$ from 4.236 to 4.663 eV and $E_{opt}^{Di}$ from 4.638 to 5.291 eV. This behavior suggests that the measured enhancement in the optical energy gap of the Tm^3+^ ion is due to a structural modification.

The observed reduction in the optical band gap may be associated with modifications of the local electronic structure and changes in the glass network. The optical energy gap is primarily determined by the energy level of the upper valence band edge, which depends on the separation of the oxygen-related states. The lower optical band-gap value observed for the Eu^3+^-doped glass may indicate increased electronic delocalization or modifications of the local bonding environment.

The higher optical band gap observed for the Tm^3+^-doped glass may be consistent with a different modification of the glass network and local electronic environment.

Occupation of such sites strengthens the cross-linking density of the glass network, reduces the NBO population, and consequently leads to higher values of both $E_{opt}^{In}$ and $E_{opt}^{Di}$.

**Figure 7:**
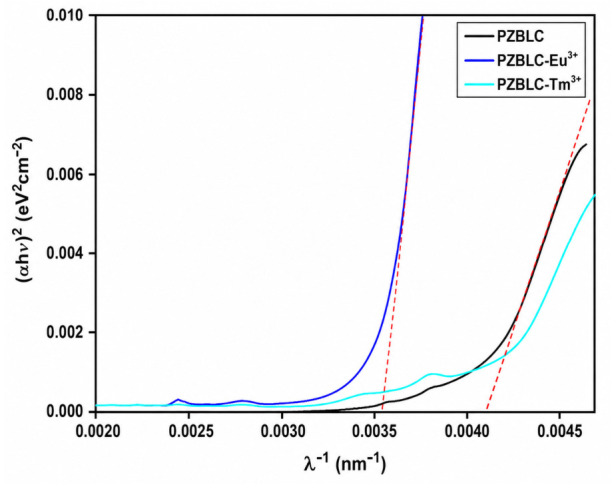
Variation of (aλ^−1^)^0.5^ vs. (λ)^−1^ of fabricated glasses.

**Figure 8:**
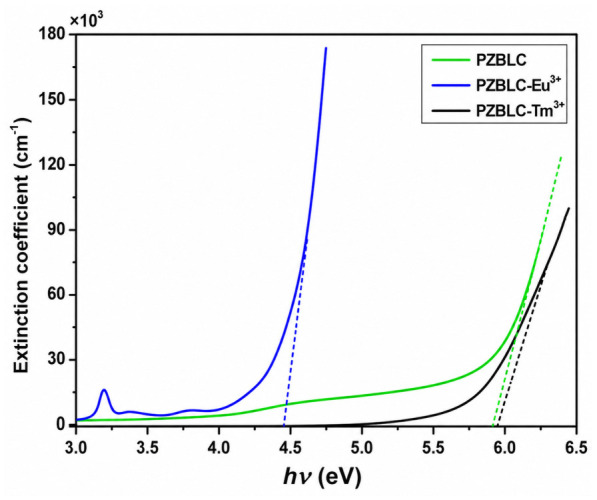
Variation in Extinction coefficient (*k*) vs. hυ (eV) of fabricated glasses.

Furthermore, the absorption spectrum fitting (ASF) method proposed by Escobar-Alarcón et al. [38] and Souri and Shomalian [39] was applied, in which the optical energy gap $E_{\mathrm{opt}}^{\mathrm{ASF}}$ can be calculated directly from the absorption spectra without requiring knowledge of the sample thickness or preparation as a thin film. For all samples, $E_{\mathrm{opt}}^{\mathrm{ASF}}$ is determined using the following equation:

(3) $$E_{opt}^{asf}=\frac{1240}{\lambda_{g}}$$

where $\lambda_{g}$ represents the wavelength corresponding to $E_{opt}^{asf}$. Figure 7 exhibits the plots of $({\frac{a}{\lambda })}^{1/2}$ versus $\lambda^{-1}$ (with a denoting absorbance) used to estimate for $E_{opt}^{asf}\;$ by extrapolating the linear portion of the curves to $\left(a/\lambda {)}^{1/2}=0\right.$. The resulting values of $1/\lambda_{g}$, λ_g_, and $E_{opt}^{asf}\;$ (in eV) for all synthesized glasses, obtained via the ASF method, are summarized in Table 3. Following this, the relationship between the absorption coefficient (α) and the extinction coefficient (κ) was employed as described in [40]:

(4) $$\alpha \;=\frac{4\pi \kappa }{\lambda }$$

the experimental optical energy gap $E_{opt}^{k}$ for all three samples was estimated via extrapolation of the linear portion of the κ versus photon energy (hν) plot to κ=0. Figure 8 shows the κ versus hν plots for all three glasses, and the corresponding $E_{\mathrm{opt}}$ values are summarized in Table 3. In conclusion, the values of $E_{\mathrm{opt}}^{\mathrm{ASF}}$ and $E_{\mathrm{opt}}^{k}$, for the fabricated glasses, follow the same trend as $E_{\mathrm{opt}}^{\mathrm{In}}$ and $E_{\mathrm{opt}}^{\mathrm{Di}}$ (see Table 3). The fabricated glass with composition 40P_2_O_5_-30ZnO-10BaF_2_-18LiCl-2.0CdO has the highest optical energy gap, making it a superior medium for doping with rare-earth ions to obtain a solid-state laser source. For numerous amorphous semiconductors, the absorption coefficient (α) exhibits an exponential increase with photon energy (hν) near the optical absorption edge, following the Urbach rule [41]:

(5) $$\alpha \left(\nu \right)=\alpha_{0}exp\;\left(\frac{h\nu }{\Delta E}\;\right),$$

(6) $$ln\alpha (\nu )=(h\nu /\Delta E)+ln(\alpha_{0})$$

**Figure 9:**
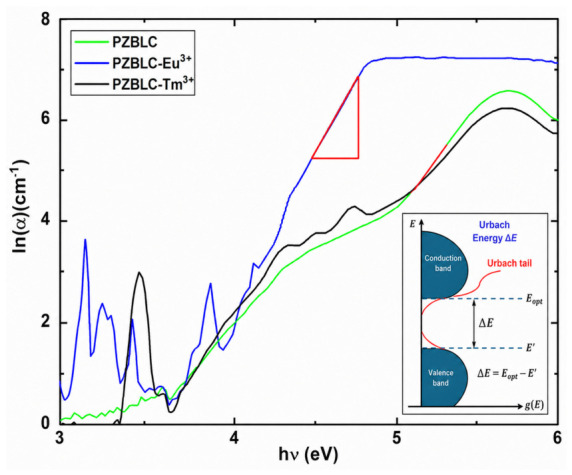
Plot of ln(α) vs. hυ of fabricated glasses.

In this expression, α_0_ is a constant, and ΔE represents the Urbach energy, which quantifies the width of the band tails associated with localized states within the band gap. This parameter reflects transitions involving localized tail states adjacent to the valence and conduction bands that extend into the band gap. ΔE can be determined from the inverse of the slopes of the linear regions in plots of ln(α) versus hν. Figure 9 presents the Urbach plots for all three glass samples, and the corresponding ΔE values obtained from these plots are summarized in Table 3.

As is well established, the Urbach energy provides insight into the degree of structural order or disorder in amorphous materials. The observed decrease in Urbach energy (ΔE = 0.155 eV) for the PZBLC–Eu^3+^ glass can be attributed to a reduction in network depolymerization caused by Eu_2_O_3_ doping, which in turn lowers the extent of structural disorder and the concentration of lattice defects. In contrast, the PZBLC–Tm^3+^ glass exhibits an increased Urbach energy (ΔE = 0.288 eV) upon incorporation of Tm_2_O_3_, indicating an increase in the degree of disorder within the glass matrix. These variations depend on the type, nature, and concentration of the introduced trivalent cations (Eu_2_O_3_ or Tm_2_O_3_), which modify the glass structure and lead to localized band-tail states in the optical gap, thereby altering the value of ΔE. The Urbach energy results indicate differences in the degree of structural disorder introduced by Eu^3+^ and Tm^3+^ ions. However, owing to the absence of direct structural characterization, these changes cannot be unambiguously correlated with specific variations in NBO concentration or network connectivity. The observed variations are therefore discussed in terms of modifications of the local electronic and structural environment induced by the rare-earth ions.

In glassy materials, the refractive index is a key parameter that critically affects the performance and reliability of optical devices. In the present study, the refractive index of the glasses synthesized was estimated using the models proposed by Moss [42], Dimitrov–Sakka (D–S) [41], and Reddy et al. [43]. The empirical relationships linking the refractive index to the optical band gap energy are expressed as follows:

Moss model:

(7) $$n_{mos}={\left(\frac{95}{E_{opt}^{In}}\right)}^{0.25}$$

Dimitrov−Sakka Model:

(8) $$n_{DS}={\left(6\sqrt{\frac{5}{E_{opt}^{In}}-2}\right)}^{0.5}$$

Reddy Model∶

(9) $$n_{RD}={\left(1+{\left(\frac{13.6}{E_{opt}^{In}+3.4}\right)}^{2}\right)}^{0.5}$$

**Figure 10:**
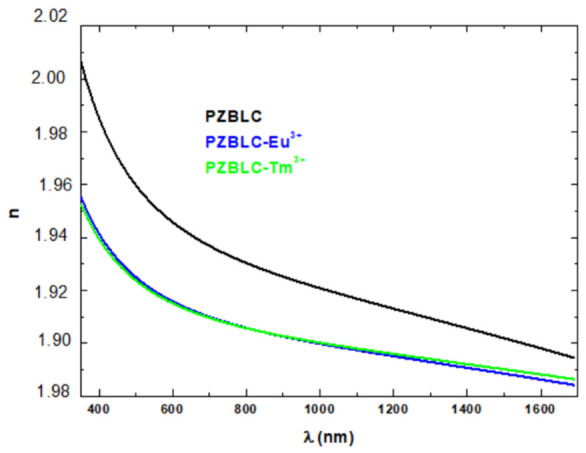
Refractive index dispersion of Eu^3+^ and Tm^3+^-doped glass samples in the BZBLC system.

The refractive index values obtained from the experimental measurements $n_{Dm}$ and the theoretical models, namely the Moss model $n_{mos}$, Dimitrov–Sakka model $n_{DS}$, and Reddy model $n_{RD}$, are presented in Table 4. The values, $n_{mos}$, $n_{DS}$, and $n_{RD}$ of PZBLC, PZBLC-Eu^3+^ and PZBLC-Tm^3+^ follow the same trend across all samples (see Table 4). Among the fabricated glasses, PZBLC–Eu^3+^ exhibits the highest refractive index ($n_{\mathrm{DS}}=2.126$), whereas the undoped PZBLC glass has the lowest value ($n_{\mathrm{DS}}=2.039$). This behavior is generally observed to follow an inverse trend with the optical energy gap. The refractive index of a glass is affected by several factors, including its density, the polarizability of nearest-neighbor anions, the coordination number of the cations, the intrinsic electronic polarizability of oxide ions, and the optical basicity of the glass network. The experimentally measured refractive indices for PZBLC, PZBLC–Eu^3+^ and PZBLC–Tm^3+^ glasses were found to be 1.946, 1.916, and 1.910, respectively (Figure 10). In contrast, the theoretical models generally predicted higher refractive-index values.

The differences between the measured refractive index ($n_{Dm}$) and the theoretical values ($n_{RD}$, $n_{DS}$, $n_{mos}$) arise because each model is based on different assumptions about the electronic structure and optical behavior of the glass [44].

The measured refractive index reflects the real optical response of the glass network. A noticeable difference between the experimentally measured and theoretically estimated refractive indices was observed for all investigated glass samples. Among the theoretical approaches, the Moss model yielded the highest refractive-index values, ranging from 2.115 to 2.176. This overestimation can be attributed to the fundamental assumptions of the Moss relation, which correlates the refractive index with the optical band gap. The Moss model was originally developed for crystalline semiconductors and assumes that the electronic polarization is primarily governed by the band-gap energy. However, phosphate-based glasses possess a highly disordered amorphous structure containing non-bridging oxygens, mixed ionic environments, and local structural heterogeneity. The Dimitrov–Sakka model produced comparatively lower values than the Moss model but still overestimated the measured refractive index. This model relates the refractive index to oxide-ion polarizability and optical band gap. Since phosphate glasses containing halides such as BaF_2_ and LiCl exhibit complex mixed-anion environments, the assumption of uniform electronic polarization becomes less accurate. Furthermore, the incorporation of Eu^3+^ and Tm^3+^ ions introduces localized 4f electronic states and modifies the local field environment, which cannot be completely accounted for within the simplified theoretical framework. Among all theoretical approaches, the Reddy model exhibited the closest agreement with the experimental refractive-index values. For the undoped PZBLC glass, the calculated value exactly matched the experimental result ($n_{RD}$ = $n_{Dm}$ = 1.946), while only small deviations were observed for the rare-earth-doped samples. The improved agreement is likely due to the semi-empirical nature of the Reddy model, which incorporates parameters associated with glass density, molar volume, and structural disorder, making it more suitable for amorphous phosphate glass systems. The lower experimental refractive-index values compared with the theoretical predictions can also be explained by the presence of halide components (BaF_2_ and LiCl), which reduce the overall electronic polarizability of the glass matrix.

In our previous studies [39,40,41], the Judd–Ofelt (JO) theory was successfully employed to determine the spectroscopic parameters, Ω_λ_ (λ = 2, 4, 6), for rare-earth-ion-doped glass matrices. In the work reported here, these parameters were determined for PZBLC–Eu^3+^ and PZBLC–Tm^3+^ glasses, which depend on both the type of rare-earth ion and the host glass composition [45]. The results presented in Table 5 and Table 6 show the oscillator strengths of Eu^3+^ and Tm^3+^ ions in the investigated glass matrix. The obtained values were determined using the same methodology as that reported in Ref. [46].

For PZBLC–Eu^3+^ glasses, the JO parameters follow the trend Ω_2_ > Ω_4_ > Ω_6_, whereas for PZBLC–Tm^3+^ glasses, the trend is Ω_2_ > Ω_4_ > Ω_6_ the same [47,48]. The relatively large value of Ω_2_ in PZBLC–Tm^3+^ indicates a stronger covalent character of Tm–O bonds and lower site symmetry around the Tm^3+^ ions. Furthermore, the Ω_2_ value of PZBLC–Eu^3+^ is larger than that of PZBLC–Tm^3+^, suggesting that Eu–O bonds exhibit a stronger covalent character within the same host glass matrix. For sample PZBLC-Eu^3+^, the highest oscillator strengths are observed for the hypersensitive transitions: ^7^F_0_ → ^5^L_6_ and ^7^F_0_ → ^5^D_2,_ indicating an asymmetric and partially covalent local environment around the Eu^3+^ ions in the glass matrix.

Based on the presented experimental ($f_{exp}$) and calculated ($f_{cal}$) oscillator strengths for the electronic transitions of Eu^3+^ ions in the investigated glasses, an excellent agreement between the experimental and theoretical oscillator strength values can be observed. This finding confirms the validity of the applied Judd–Ofelt (J–O) model as well as the high quality of the intensity parameter fitting procedure. The differences between the $f_{exp}$ and $f_{cal}$ values remain very small for all analyzed transitions, indicating that the obtained Ω_2_, Ω_4_, and Ω_6_ parameters accurately describe the optical behavior of the Eu^3+^ ion system embedded in the PZBLC glass matrix. Particularly good agreement is observed for the intense absorption transition: ^7^F_0_- > ^5^L_6,_ for which the oscillator strengths $f_{exp}=8.50\times 10^{-6}$ and $f_{cal}=8.00\times 10^{-6}$ indicate only a minor fitting deviation and therefore demonstrate the high reliability of the theoretical calculations. This transition exhibits the highest oscillator strength among all analyzed transitions. Similar behavior has been reported for modern lanthanide-doped phosphate glasses, where intense electric-dipole transitions were attributed to enhanced asymmetry of the local ligand field and to the partially covalent character of RE–O bonds [49].

The presence of phosphate and halide constituents within the P_2_O_5_–ZnO–BaF_2_–LiCl–CdO glass matrix contributes to the reduction in the phonon energy of the host material, thereby suppressing non-radiative relaxation processes and enhancing luminescence efficiency. Such low-phonon glass systems are considered particularly favorable for rare-earth-doped photonic applications due to their ability to improve radiative emission probabilities and prolong excited-state lifetimes. Furthermore, the literature emphasizes that Judd–Ofelt parameters are highly sensitive to the local chemical structure of the glass network [50]. Studies on rare-earth-doped phosphate glasses have demonstrated that an increase in the asymmetry surrounding rare-earth ions leads to higher Ω_2_ values and enhanced hypersensitive transition intensities. An analogous phenomenon can be observed in the investigated PZBLC glass system, where the relatively high oscillator strengths of Eu^3+^ transitions suggest that europium ions occupy sites of low local symmetry and are strongly coupled to the glass network.

**Figure 11:**
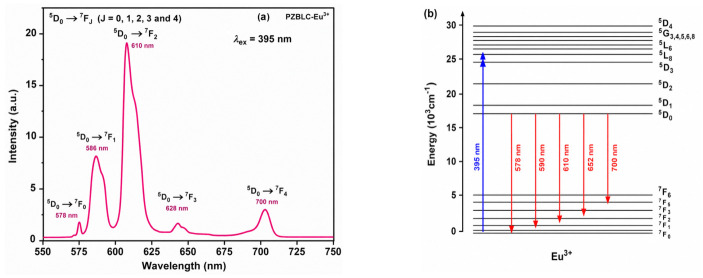
Visible emission spectra of rare earth doped samples (**a**) PZBLC-Eu^3+^, (**b**) Energy diagram of PZBLC-Eu^3+^.

The room-temperature emission spectrum of the fabricated PZBLC–Eu^3+^ glass is shown in Figure 11a. The spectrum exhibits characteristic Eu^3+^ emission bands corresponding to the ^5^D_0_ → ^7^F_J_ transitions (J = 0, 1, 2, 3, 4), specifically: ^5^D_0_ → ^7^F_0_ (578 nm), ^5^D_0_ → ^7^F_1_ (590 nm), ^5^D_0_ → ^7^F_2_ (610 nm), ^5^D_0_ → ^7^F_3_ (652 nm), and ^5^D_0_ → ^7^F_4_ (700 nm). These transitions are attributed to Eu^3+^ ions in the glass matrix. The corresponding energy-level diagram of these transitions is presented in Figure 11b. As a result, the PZBLC–Eu^3+^ glasses exhibit intense red luminescence at room temperature. We note that the fabricated glass, PZBLC-Eu^3+^, exhibits intense brightness and orangish-red luminescence at 590–720 nm, corresponding to the ^5^D_0_ → ^7^F_2_ transition. This material may find potential applications in LEDs and field-emission devices, owing to its high luminescence efficiency, characterized by narrow monochromatic emission around 610 nm and a long radiative lifetime of PZBLC–Eu^3+^ (τ= 2590 μs, see Figure 13). These fabricated glasses may be used in therapeutic and clinical applications, as they exhibit maximal absorption bands suitable for photosensitizer activation.

The visible luminescence emission spectrum of the $Eu^{3+}$ ion is characterized by the transitions D0 5 →Fj 7 (j = 0 to 6) from the D0 5 exited state to the J levels of the ground state term Fj 7. In particular, the most intense emission peak is around 610 nm (electrical dipole transition D0 5 →F2 7 that ensures a red luminescence. The magnetic dipole transition D0 5 →F1 7 (590 nm) is independent of the symmetry of the local environment. Many studies have investigated the factors influencing specific luminescence in glasses, particularly the role of glass composition [51]. These studies concluded that incorporating cations of lighter elements into the host glass enhances the red fluorescence associated with the ^5^D_0_ → ^7^F_2_ transition by facilitating cross-relaxation processes between Eu^3+^ ions. However, at high Eu^3+^ concentrations, these same processes can lead to fluorescence quenching. Babu et al. [16] investigated the optical absorption and photoluminescence behavior of Eu^3+^ ions in phosphate and fluorophosphate glasses. They found that a larger value of the intensity parameter Ω_2_ signifies the hypersensitive character of the ^5^D_0_ → ^7^F_2_ transition, suggesting that Eu^3+^ ions reside in a highly polarizable environment within the glass matrix. Furthermore, the addition of BaF_2_ to the phosphate glass reduced the non-radiative decay rate, causing a slight enhancement in quantum efficiency. However, modifying the phosphate glass with BaF_2_ did not result in a significant change in the experimentally measured lifetime.

In fluoroaluminate glass matrices, the highly ionic character imparted by fluorine ligands has been observed to stabilize the Eu^3+^ ions, resulting in an extended fluorescence lifetime of approximately 7.0 ms [52]. Increasing the concentrations of $Eu^{3+}$ in phosphate and zinc fluorophosphate glasses might increase the intensity of absorption and emission, while the fluorescence lifetime or decay rate was independent of $Eu^{3+}$ concentrations, although it does depend on the host material and the probability of non-radiative energy transfer between $Eu^{3+}$ ions [17,52].

**Figure 12:**
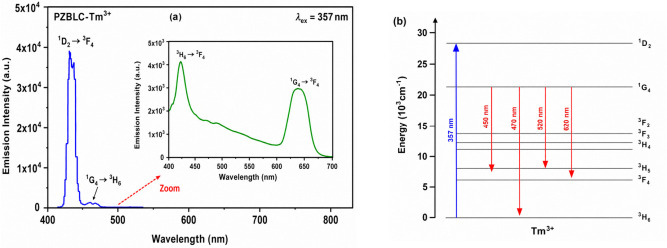
Visible emission spectra of rare earth doped samples (**a**) PZBLC-Tm^3+^, (**b**) Energy diagram of PZBLC-Tm^3+^.

The photoluminescence (PL) emission characteristics of the fabricated PZBLC glasses doped with Tm^3+^ ions, shown in Figure 12a, were measured at an excitation wavelength of 357 nm, revealing four emission bands as follows: two in blue ^1^D_2_ → ^3^F_4_ (450 nm) and ^1^G_4_ → ^3^H_6_ (476 nm), green ^1^D_2_ → ^3^H_5_ (520 nm), and red ^1^G_4_ → ^3^F_4_ (660 nm). The intensity of the emission transitions (^1^G_4_ → ^3^H_6_, ^1^D_2_ → ^3^H_5_, and ^1^G_4_ → ^3^F_4_) is low compared with that of the ^1^D_2_ → ^3^F_4_ transition, but the blue emission at 450 nm is very intense. The corresponding energy-level transitions in this glass are illustrated in the energy diagram shown in Figure 12b.

**Figure 13:**
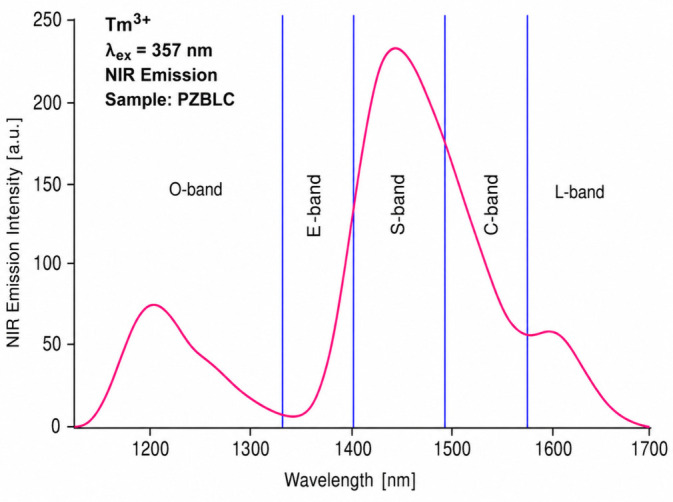
NIR-PL emission spectra of glasses PZBLC-Tm^3+^ under excitation at 357 nm.

The near-infrared (NIR) luminescence spectra of the fabricated PZBLC–Tm^3+^ glasses were recorded under excitation at 357 nm and are shown in Figure 13. A broad NIR emission band is observed in the range of 1350–1700 nm. In these glasses, the strong local field around Tm^3+^ ions leads to pronounced splitting of the ^3^H_6_ → ^3^F_4_ transition, resulting in enhanced NIR emission and further broadening of the band. Consequently, the ^3^H_6_ → ^3^F_4_ transition spans from 1.35 μm to 1.7 μm, covering the E-, S-, C-, and L-bands for optical amplification, with an effective bandwidth of Δλ_eff_ = 141 nm, making it suitable for wavelength-division-multiplexed (WDM) communication applications. Wideband fiber amplifiers offer the advantage of operating in the NIR region, with emission shifted toward longer wavelengths, covering the C and L communication bands. Consequently, the fabricated PZBLC–Tm^3+^ glasses are well-suited for WDM systems, providing broad gain bandwidths and potentially reducing overall system costs. As shown in Figure 13, a strong and broad emission is observed at 1200 nm, corresponding to the ^1^G_4_ → ^3^H_4_ transition of Tm^3+^ ions. Additional overlapping emission bands are present at approximately 1452, 1514, and 1620 nm, assigned to the ^3^H_4_ → ^3^F_4_, ^1^G_4_ → ^3^F_2_, and ^3^F_4_ → ^3^H_6_ transitions, respectively. Therefore, the synthesized Tm^3+^-doped glasses are expected to exhibit up-conversion (UC) luminescence spanning multiple spectral regions, including red emissions (1G4→3F4F33→3H6, blue emissions (1D2→3F4G41→3H6, ultraviolet emissions (1I6→3F4D21→3H6, and near-infrared (NIR) emission $\left.{(}^{3}H_{4}\to^{3}H_{6}\right)$ under visible or near-infrared excitation. Such broadband UC emissions are of considerable interest for applications in color displays, optical data storage, underwater optical communication, visible lasers, and biomedical technologies.

**Figure 14:**
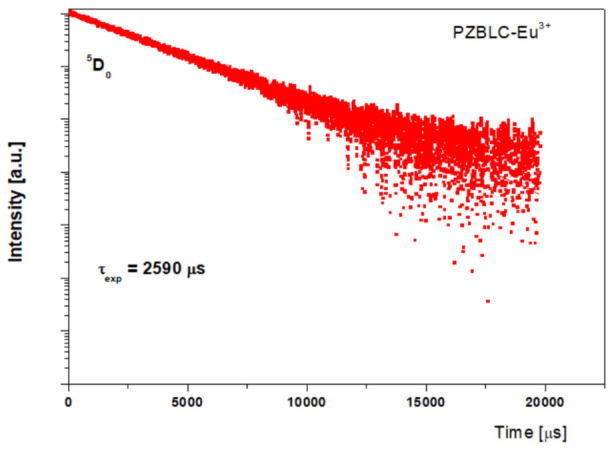
Fluorescence decay lifetime level (^5^D_0_) of glass PZBLC-Eu^3+^.

**Figure 15:**
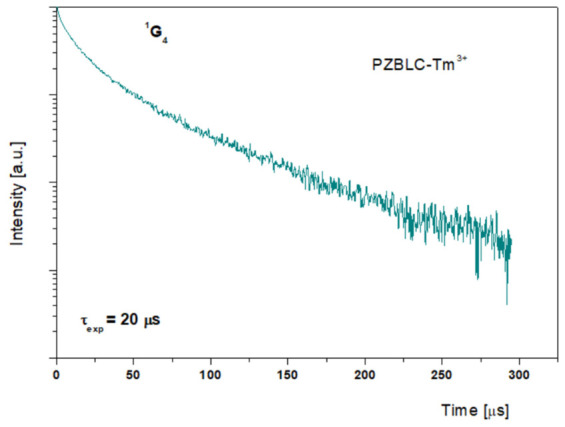
Fluorescence decay lifetime level (^1^G_4_) of glass PZBLC-Tm^3+^.

**Figure 16:**
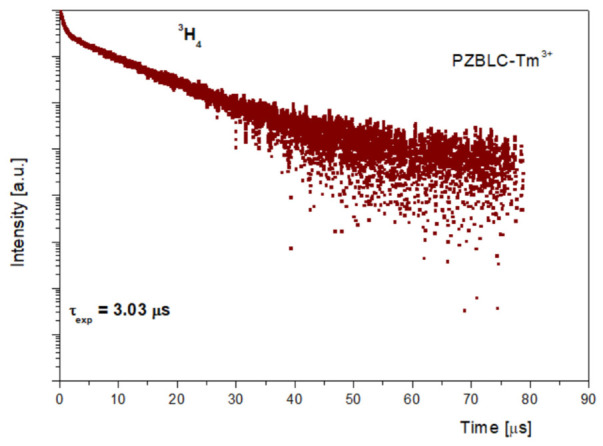
Fluorescence decay lifetime level (^3^H_4_) of glass PZBLC-Tm^3+^.

In the fabricated glasses, the experimental lifetimes were previously determined by our research group using the same methodology [29], where

(10) $$\tau_{\exp}=\int \frac{t\;\varepsilon (t)}{\varepsilon (t)}\;dt,$$

and the results are presented in Figure 14, Figure 15 and Figure 16. The radiative lifetimes estimated for the prepared glasses are as follows: τ_exp_ = 20 μs for the ^1^G_4_ level of PZBLC–Tm^3+^, τ_exp_ = 3.03 μs for the ^3^H_4_ level of PZBLC–Tm^3+^, and τ_exp_ = 2590 μs for the ^5^D_0_ level of PZBLC–Eu^3+^. It is well established that the presence of hydroxyl (OH) groups induces luminescence quenching via non-radiative relaxation pathways, which originate from O–H stretching vibrations in the frequency range of 3500–3900 cm^−1^ [53]. Consequently, lifetime measurements of the ^5^D_0_ excited state of Eu^3+^ ions can be employed to estimate the average number of water molecules coordinated in the first coordination sphere of the europium ion [54].

The radiative lifetimes were presented in Table 7 for all investigated samples. Radiative lifetimes of the (^5^D_0_) level of Eu^3+^ ions were estimated to be approximately 4 ms, which is characteristic of phosphate-based glass systems. In conjunction with the experimentally determined lifetime of 2.59 ms, this result indicates a relatively high emission quantum efficiency. Furthermore, the comparison between the experimental and radiative lifetimes suggests a limited contribution of non-radiative relaxation processes and confirms the good optical quality of the glass matrix.

The lifetime data of the excited states of Tm^3+^ ions reveal a significant contribution of non-radiative processes in the investigated glass system. This effect is particularly pronounced for the ^1^G_4_ level, for which the experimental lifetime was found to be only τ_exp_ = 20 μs, whereas the corresponding radiative lifetime was estimated to be τ_rad_ = 0.777 ms. Consequently, the emission quantum efficiency is very low, reaching only η = 2%. Such a substantial reduction in the experimental lifetime indicates the predominance of non-radiative relaxation mechanisms, including multiphonon relaxation, energy transfer between Tm^3+^ ions, concentration quenching, as well as quenching induced by OH^−^ groups present in the glass matrix. The ^1^G_4_ state is a highly excited energy level and is therefore particularly susceptible to non-radiative losses in phosphate-based glasses, which are characterized by relatively high phonon energies.

Moreover, the ^3^H_4_ level plays a crucial role in near-infrared emission processes; therefore, such pronounced quenching of this excited state may significantly limit the laser and photonic potential of the investigated Tm^3+^-doped glass system.

Phosphate-based PZBLC glasses doped with Eu^3+^ ions represent promising materials for red visible-light emission under 395 nm excitation. Moreover, the investigated system exhibits a relatively high quantum efficiency of approximately 65%, indicating efficient radiative relaxation and favorable optical quality of the host matrix.

In contrast, PZBLC glasses doped with Tm^3+^ ions, excited at 357 nm, demonstrate broadband near-infrared (NIR) emission, which is suitable for optical amplification applications. However, the experimentally determined lifetimes are significantly shorter than the radiative ones, primarily due to strong multiphonon relaxation processes within the glass matrix. As a consequence, the overall luminescence efficiency is considerably reduced, which limits their applicability as efficient standard light sources.

The absorption cross-section (σₐ) of the doped rare-earth ions can be determined from the measured absorption spectrum according to the Beer-Lambert formula.

(11) $$\sigma_{a}(\lambda )=2.303{\left(NL\right)}^{-1}log(\frac{I_{0}}{I})$$

where I_0_ is the optical intensity, and I is the optical intensity throughout the sample. The emission cross-section is an interesting parameter related to laser performance. Using the McCumber theory [5,41], the stimulated emission cross section σ_e_ can be measured using the absorption cross section as follows:

(12) $$\sigma_{e}\left(\lambda \right)=\sigma_{a}\left(\lambda \right)\cdot (Z_{l}){\left(Z_{u}\right)}^{-1}\exp\left[hc{\left(kT\right)}^{-1}(\frac{\lambda_{ZL}-\lambda }{\lambda \lambda_{ZL}})\right]$$

Here, $Z_{L}$ and $Z_{U}\;$ indicate the distribution functions of the lower and upper states, respectively, with their ratio given as 1.488. T denotes the temperature, k  is the Boltzmann constant, and $\lambda_{ZL}$, the zero-phonon line, corresponds to the wavelength associated with the transition between the lower Stark sublevels of the emitting state and those of the receiving state. In the present study, $\lambda_{ZL}$ was taken as 1767 nm for the calculation of the emission cross-section [41].

**Figure 17:**
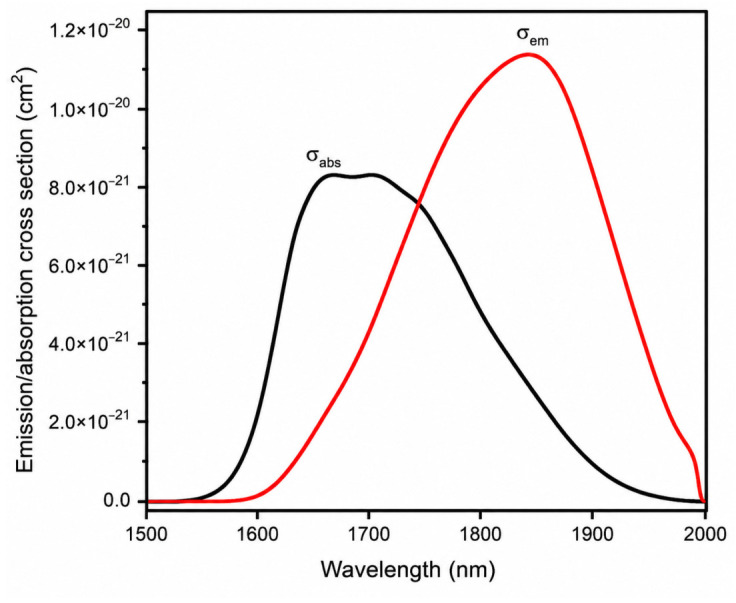
Emission cross section, σ_em_, and absorption cross section, σ_abs_, of PZBLC-Tm^3+^ glasses.

As shown in Figure 17, the maximum emission cross-section (σ_em_) of the glass was determined at 1.84 μm, yielding a value of σ_em_ = 1.18 × 10^−20^ cm^2^ with an effective bandwidth of Δλ_eff_ = 141.7 nm for the ^3^F_4_ → ^3^H_6_ transition, corresponding to near-infrared (NIR) emission. This transition is widely recognized as one of the most important radiative channels of Tm^3+^ ions for eye-safe laser systems and near-infrared photonic applications.

The emission cross-section is an important spectroscopic parameter used to evaluate the gain bandwidth (GBW = σ_em_ × Δλ_eff_), which is directly related to the tunability and amplification potential of laser materials. For the investigated glass, the gain bandwidth was calculated to be 16.7 × 10^−26^ cm^3^.

A relatively large gain bandwidth is highly desirable for tunable and ultrashort-pulse laser systems because it supports broader emission spectra and wider wavelength tuning ranges. The obtained GBW value is comparable to those reported for several Tm^3+^-doped phosphate and fluorophosphate glass systems, suggesting that the fabricated PZBLC–Tm^3+^ glass is a promising candidate for near-infrared photonic applications [55,56].

The favorable spectroscopic performance may be associated with the mixed halide–phosphate glass matrix, which combines relatively low phonon energy with good rare-earth ion solubility and high optical transparency.

In addition, the presence of BaF_2_ and LiCl in the glass composition may contribute to reduced multiphonon relaxation rates, thereby supporting enhanced radiative transitions associated with the ^3^F_4_ → ^3^H_6_ emission band of Tm^3+^ ions near 1.8 μm. This near-infrared transition is widely recognized as an important emission channel for eye-safe laser systems, optical amplifiers, and infrared sensing technologies.

**Figure 18:**
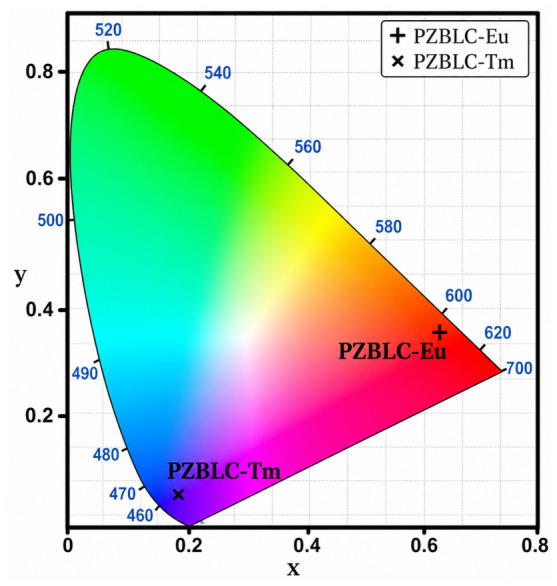
The CIE 1931 chromaticity diagram for the investigated glasses under an excitation wavelength of 357 nm (for Tm^3+^ ions) and 395 nm (for Eu^3+^ ions).

The visible luminescence properties of the glasses were further evaluated using chromaticity coordinates. For PZBLC–Eu^3+^ glasses, the coordinates were calculated from emission spectra under 395 nm excitation, while for PZBLC–Tm^3+^ glasses, 357 nm excitation was used. The emission coordinates were mapped onto the Commission Internationale de l’Éclairage (CIE) 1931 chromaticity diagram (Figure 18), indicating high color purity and well-defined emission hues. These characteristics make the fabricated glasses promising candidates for photonic applications, including solid-state lighting, display technologies, and optical signaling devices.

The determined chromaticity coordinates (x, y) for the red-emitting glasses were (0.64, 0.36), indicating an emission very close to the standard red reference. In contrast, the PZBLC-Tm^3+^ glass exhibited blue emission with coordinates of (x = 0.158, y = 0.041), demonstrating the tunable luminescence properties of the fabricated glass systems.

The CIE chromaticity coordinates confirm the generation of highly saturated red and blue emissions. Therefore, the investigated glasses may be considered promising red-and blue-emitting photonic materials.

## 4. Conclusions

A host glass 40P_2_O_5_-30ZnO-10BaF_2_-18LiCl-2.0Cd (PZBLC) has the highest density (3.2573 g/cm^3^). Incorporation of Eu^3+^ and Tm^3+^ ions into the host glass leads to an increase in thermal stability from 74 to 79 °C and glass transition temperature from 360 to 376 °C. The host glass (PZBLC) has the highest value of indirect optical gap ($E_{opt}^{In}$= 4.749 eV); otherwise, the host glass doped with Eu^3+^ ions has the lowest value ($E_{opt}^{In}$= 4.234 eV). According to the spectroscopic ellipsometry analysis, the refractive index of the Eu^3+^-doped glass reached n_RD_ = 2.04. Overall, the obtained results demonstrate that although theoretical models provide useful insight into the optical behavior of halide phosphate glasses, the experimentally measured refractive index remains the most reliable parameter for practical optical characterization. Among the investigated models, the Reddy model provides the best approximation for the PZBLC glass system. Moreover, the PZBLC-Eu^3+^ glass exhibits a low Urbach energy (ΔE = 0.155 eV), indicating reduced depolymerization, lower structural disorder, and fewer lattice defects in the fabricated glass. In contrast, the PZBLC-Tm^3+^ glass exhibits favorable spectroscopic properties, with a gain bandwidth (GBW = σ_em_ × Δλ_eff_) of 16.7 × 10^−26^ cm^3^. The combination of a broad emission band and a relatively high emission cross-section suggests its potential for near-infrared optical applications. In addition, the PZBLC-Eu^3+^ glass shows promising red emission under 395 nm excitation. The Eu^3+^-doped phosphate-based PZBLC glasses exhibit considerable potential as red-emitting materials under 395 nm excitation. Furthermore, the investigated glass system demonstrates a relatively high quantum efficiency of approximately 65%, indicating efficient radiative relaxation processes and favorable optical quality of the host matrix. These characteristics highlight the suitability of the developed glasses for visible-light photonic applications and red-light-emitting devices. In contrast, the PZBLC-Tm^3+^ glasses, excited at 357 nm, exhibit broadband near-infrared (NIR) emission suitable for optical amplification. Additionally, these glasses emit blue light, making them useful as standard light sources. The structural interpretations proposed in this work are based on indirect evidence derived from density, thermal, optical and spectroscopic measurements. Since no direct structural characterization (FTIR, Raman, XPS, MAS-NMR or EXAFS) was performed, the proposed changes in NBO concentration, network connectivity and possible P–O–RE interactions should be considered as plausible interpretations rather than direct confirmation. Future studies will focus on advanced structural investigations to verify these hypotheses.

## Figures and Tables

**Table 1:** The density (ρ) molar volume ($V_{m\;}$), oxygen molar volume ($V_{ο}$), Oxygen packing density (OPD), molar refractivity, $R_{m}$, and molar polarizability$,\;\alpha_{m}$.

Sample	ρ $\left(g\;{cm}^{-3}\right)$	$V_{m}$ (${cm}^{3}$ ${mol}^{-1}$)	$V_{ο}$ (${cm}^{3}$ ${mol}^{-1}$)	OPD (mol $L^{-1}$)	*(R_m_)* m^3^/mol	α_m_ Å^3^
PZBLC	3.26	33.44	14.41	69.38	17.15	6.80
PZBLC-${Eu}^{3+}$	3.10	39.05	16.1	62.097	21.08	8.37
PZBLC-${Tm}^{3+}$	3.13	39.17	16.15	61.92	20.25	8.04

**Table 2:** The glass transition temperature ($T_{g\;}$), glass onset temperature ($T_{x\;}$), and thermal stability (∆T) of fabricated glasses.

Sample	$T_{g}$	$T_{x}$	∆T	C_p_ (J·mol^−1^·K^−1^)
PZBLC	360 ± 3	434 ± 3	74	0.268
PZBLC-${Eu}^{3+}$	372 ± 3	450 ± 3	78	0.237
PZBLC-${Tm}^{3+}$	376 ± 3	455 ± 3	79	0.361

**Table 3:** Direct optical energy gap, $E_{opt}^{Di}$, indirect optical energy gap, $E_{opt}^{In}$, $E_{opt}^{ASF}$, and $E_{opt}^{k}$ in eV of fabricated glasses.

Sample	Direct Optical Band Gap $E_{opt}^{Di}\;(eV)$	Indirect Optical Band Gap $E_{opt}^{In}\;(eV)$	Optical Band Gap $E_{OPT}^{ASF}$ (eV)	$E_{opt}\;from$ “k” $E_{opt}^{k}\;(eV)$	ΔE (eV)
PZBLC	5.322	4.749	5.145	5.108	0.226
PZBLC-${Eu}^{3+}$	4.638	4.236	4.460	4.503	0.155
PZBLC-${Tm}^{3+}$	5.291	4.663	5.123	5.065	0.288

**Table 4:** Linear refractive index, Moss model, n_mos_, Dimitrov–Sakka Model, n_DS_, Reddy Model, n_RD_, $n_{Dm}$-measured spectroscopic parameters, Ω_λ_ (λ = 2, 4, 6), the value of the root mean square deviation δ_RMS_ of fabricated glasses.

Sample Code	$n_{mos}$	$n_{DS}$	$n_{RD}$	$n_{Dm}$	$\Omega_{2}(pm^{2})$	$\Omega_{4}(pm^{2})$	$\Omega_{6}(pm^{2})$	*δ_RMS_*
PZBLC	2.12 ± 0.10	2.04 ± 0.07	1.95 ± 0.09	1.950 ± 0.006	none	none	none	none
PZBLC-${Eu}^{3+}$	2.18 ± 0.10	2.13 ± 0.07	2.04 ± 0.09	1.920 ± 0.002	6.043 ± 0.003	3.098 ± 0.043	2.975 ± 0.001	10.1
PZBLC-${Eu}^{3+}$	2.13 ± 0.10	2.05 ± 0.07	1.96 ± 0.09	1.910 ± 0.004	4.061 ± 0.002	2.200 ± 0.023	1.503 ± 0.003	12.35

**Table 5:** Experimental and calculated values of the oscillator of electron transition of Eu^3+^ ions in PZBLC glasses from the P_2_O_5_–ZnO–BaF_2_–LiCl–CdO system.

Transition	λ (nm)	E (eV)	Oscillator Strength *f_exp_* (×10^−6^)	Oscillator Strength f_cal_ (×10^−6^)
			BZBLC-Eu^3+^	
^7^F_0_ → ^5^H_3_	318	3.90	1.200±0.001	1.000±0.003
^7^F_0_ → ^5^D_4_	362	3.42	2.100 ± 0.023	1.900±0.034
^7^F_0_ → ^5^G_4_	382	3.25	3.500±0.021	3.200±0.004
^7^F_0_ → ^5^L_6_	394	3.15	8.500±0.011	8.000±0.005
^7^F_1_ → ^5^D_3_	414	2.99	1.700±0.002	1.500±0.003
^7^F_0_ → ^5^D_2_	464	2.68	0.6100±0.0003	0.5600±0.0002
^7^F_1_ → ^5^D_1_	534	2.32	0.0080±0.0001	0.0070 ± 0.0012
^7^F_0_ → ^7^F_6_	2084	0.59	0.015 ± 0.002	0.012 ± 0.001
^7^F_1_ → ^7^F_6_	2206	0.56	0.0110 ± 0.0001	0.0100±0.0001

**Table 6:** Experimental and calculated values of the oscillator strength of the electron transition of Tm^3+^ ions in PZBLC glasses from the P_2_O_5_–ZnO–BaF_2_–LiCl–CdO system.

Tran. ^3^H_6_→	λ (nm)	E (eV)	Oscillator Strength *f*_exp_ (×10^−6^)	Oscillator Strength *f*_cal_ (×10^−6^)
	BZBLC-Tm^3+^			
^1^D_2_	358	3.46	4.50±0.03	4.20 ± 0.05
^1^G_4_	474	2.62	3.20 ± 0.02	3.00 ± 0.06
^3^F_2_	660	1.88	1.10 ± 0.05	1.00 ± 0.02
^3^F_3_	686	1.81	0.90 ± 0.01	0.85 ± 0.01
^3^H_4_	792	1.57	2.50 ± 0.05	2.30 ± 0.01
^3^H_5_	1210	1.02	0.35000±0.00023	0.31000±0.00045
^3^F_4_	1712	0.72	0.18000 ± 0.00012	0.17000±0.00012

**Table 7:** Lifetimes of the Eu^3+^ and Tm^3+^ emission bands in halidephosphate glasses determined experimentally (τ_exp_) and those calculated within the Judd-Ofelt approach (τ_rad_), along with quantum efficiencies (η = τ_exp_/τ_rad_).

Sample	^5^D_0_ Lifetime Eu^3+^			^1^G_4_ Lifetime of Tm^3+^			^3^H_4_ Lifetime of Tm^3+^		
	τ_exp_ (μs)	τ_rad_ (ms)	η (%)	τ_exp_ (μs)	τ_rad_ (ms)	η (%)	τ_exp_ (μs)	τ_rad_ (ms)	η (%)
PZBLC-Eu^3+^	2590	4.0	65	-	-	-	-	-	-
PZBLC-Tm^3+^	-	-	-	20	0.777	2	3.03	2.83	0.1

## Data Availability

The original contributions presented in this study are included in the article. Further inquiries can be directed to the corresponding author.
